# Intra-ER sorting of the peroxisomal membrane protein Pex3 relies on its luminal domain

**DOI:** 10.1242/bio.20134788

**Published:** 2013-06-25

**Authors:** Mohammad H. Fakieh, Peter J. M. Drake, Joanne Lacey, Joanne M. Munck, Alison M. Motley, Ewald H. Hettema

**Affiliations:** Department of Molecular Biology and Biotechnology, University of Sheffield, Western Bank, Sheffield S10 2TN, UK

**Keywords:** Peroxisome, Peroxin, Pex3, Peroxisomal ER, Intra-ER sorting

## Abstract

Pex3 is an evolutionarily conserved type III peroxisomal membrane protein required for peroxisome formation. It is inserted into the ER membrane and sorted via an ER subdomain (the peroxisomal ER, or pER) to peroxisomes. By constructing chimeras between Pex3 and the type III ER membrane protein Sec66, we have been able to separate the signals that mediate insertion of Pex3 into the ER from those that mediate sorting within the ER to the pER subdomain. The N-terminal 17-amino acid segment of Pex3 contains two signals that are each sufficient for sorting to the pER: a chimeric protein containing the N-terminal domain of Pex3 fused to the transmembrane and cytoplasmic segments of Sec66 sorts to the pER in wild type cells, and does not colocalise with peroxisomes. Subsequent transport to existing peroxisomes requires the Pex3 transmembrane segment. When expressed in *Drosophila* S2R+ cells, ScPex3 targeting to peroxisomes is dependent on the intra-ER sorting signals in the N-terminal segment. The N-terminal segments of both human and *Drosophila* Pex3 contain intra-ER sorting information and can replace that of ScPex3. Our analysis has uncovered the signals within Pex3 required for the various steps of its transport to peroxisomes. Our generation of versions of Pex3 that are blocked at each stage along its transport pathway provides a tool to dissect the mechanism, as well as the molecular machinery required at each step of the pathway.

## Introduction

**Fig. 1.**
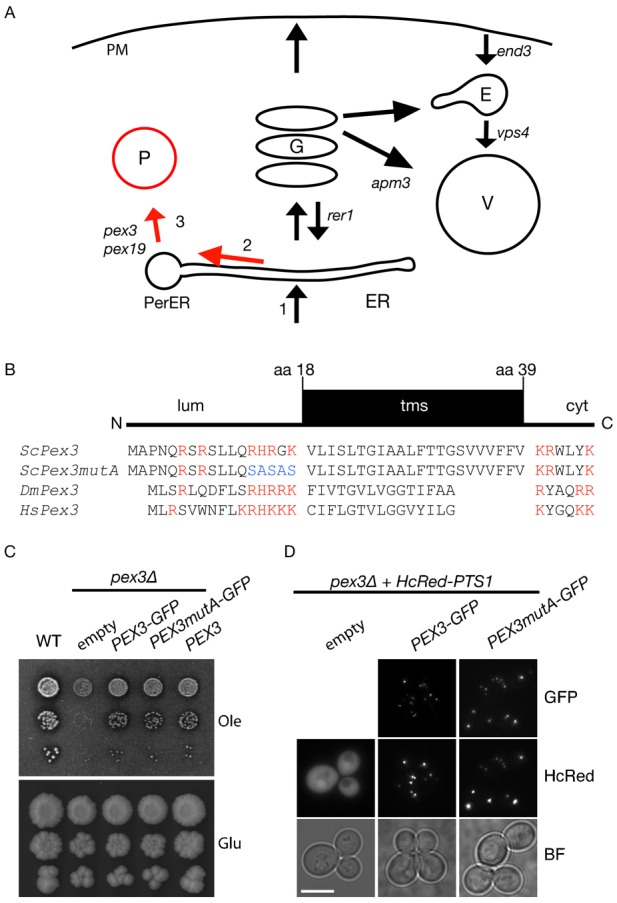
The basic cluster in the N-terminal segment of ScPex3 is not required for function. (**A**) Trafficking pathways discussed in the study. (1) Integral membrane proteins insert into the ER membrane and either follow the secretory pathway or are diverted to ER subdomains. Pex3 requires its (2) N-terminal segment to sort to the peroxisomal ER subdomain and (3) its transmembrane segment to reach peroxisomes. Mutants that block membrane trafficking at various stages are indicated. PerER, Peroxisomal ER subdomain; P, peroxisome; G, Golgi; E, endosomes; V, vacuole; PM, plasma membrane. (**B**) Schematic representation of the Pex3 targeting domain including amino acid sequences of Pex3 orthologues used in this study. N, amino-terminus; lum, luminal segment; tms, transmembrane segment; cyt, cytosolic segment. aa 18, amino acid residue number 18. Basic residues indicated in red. Mutation of basic cluster indicated in blue. *Sc, Saccharomyces cerevisiae; Dm, Drosophila melanogaster; Hs, Homo sapiens*. (**C**) The basic cluster is not required for Pex3 function. Pex3 and Pex3mutA tagged with GFP and expressed from Pex3 promoter complement the growth defect of *pex3*Δ cells on oleate medium (Ole). Normal growth on glucose medium is observed in all strains. (**D**) Both Pex3-GFP versions restore peroxisome formation and colocalise with the peroxisomal luminal marker HcRed-PTS1. Scale bar: 5 µm.

Membrane proteins of the endomembrane system are first inserted into the ER before they are sorted to their cellular destinations. After entry into the ER, most of these proteins follow the secretory pathway (Fig. 1A), i.e. are incorporated into COPII transport vesicles and are transported to the Golgi. Many of these proteins have specific signals that bind either directly or via a cargo receptor to COPII components. Additional signals may be required to sort proteins to the various compartments of the endocytic system or to retain them in secretory compartments. Some proteins are sorted to subdomains of the ER or to compartments that originate from this organelle, for example, lipid bodies and peroxisomes (Lynes and Simmen, 2011). Intra-ER sorting is a poorly understood process but signals and protein elements that mediate these sorting processes are being identified (Castillon et al., 2009; Ingelmo-Torres et al., 2009; Lynes and Simmen, 2011; Ronchi et al., 2008; Watanabe and Riezman, 2004).

Peroxisomal membrane proteins (PMPs) can be classified based on their requirement for Pex19 binding for delivery to peroxisomes (Jones et al., 2004; Matsuzono and Fujiki, 2006; Pinto et al., 2006; for a review, see Rucktäschel et al., 2011). A small number of PMPs, including Pex3 and Pex22, contain a targeting domain that is not recognized by Pex19 (Halbach et al., 2009). Pex3 and Pex22 both have type III topology and they both traffic via a subdomain of the ER known as the preperoxisomal compartment or the peroxisomal ER (pER), to peroxisomes. The N-terminal domain of Pex3 (aa1–45 of ScPex3), consisting of the luminal segment containing an evolutionarily conserved basic cluster followed directly by its transmembrane segment plus a few amino acids of the cytoplasmic domain, is necessary and sufficient for its transport to peroxisomes in plants, fungi and animals (Baerends et al., 2000; Hunt and Trelease, 2004; Kammerer et al., 1998; Soukupova et al., 1999; Tam et al., 2005; Wiemer et al., 1996). Substitution of the cluster of basic residues results in Pex3 mislocalisation (Baerends et al., 2000; Hunt and Trelease, 2004). However, its precise role remains unclear. Sec61 has been implicated in peroxisome formation, and it has been proposed that targeting of Pex3 to the ER depends on a signal-anchor-like sequence consisting of the evolutionarily conserved cluster of basic amino acid residues in the N-terminal segment followed by a hydrophobic transmembrane segment (Thoms et al., 2012) (Fig. 1B). The signal(s) in Pex3 that mediate its subsequent sorting to the ER subdomain and to peroxisomes remain unclear. Peroxisome biogenesis, a process that requires Pex3 sorting, occurs independently of COPI or COPII (Lam et al., 2010; South et al., 2000; Voorn-Brouwer et al., 2001).

A role for a subdomain of the ER in peroxisome formation became clear in *S. cerevisiae* cells temporarily devoid of peroxisomes, when peroxisomes must form *de novo* (Hoepfner et al., 2005; Tam et al., 2005; van der Zand et al., 2010). Some newly synthesised PMPs, including Pex3, concentrate in this ER subdomain and with time these structures lose their association with the ER and mature into new peroxisomes by acquisition of additional membrane and matrix proteins. In cells containing peroxisomes, these ER-derived carriers fuse with existing peroxisomes (Motley and Hettema, 2007).

However, whether peroxisomes form de novo in wild type yeast cells or whether they multiply by growth and division remains contentious (Nuttall et al., 2011). Recently, it has been postulated that all PMPs in *S. cerevisiae* travel via the ER. Van der Zand et al. propose that PMPs segregate in the ER and exit the ER in distinct vesicles. These post-ER carriers are proposed to fuse to form new functional peroxisomes in wild type cells, contributing to the existing population of peroxisomes (van der Zand et al., 2010; van der Zand et al., 2012).

We reinvestigated the biogenesis of Pex3 with emphasis on the identification of signals that direct its transport to peroxisomes. We found that, in contrast to earlier suggestions, Pex3 is targeted and inserted into the ER by a sequence consisting of its transmembrane segment followed by a short cytoplasmic region. We show that the N-terminal 17 amino acids of ScPex3 contains two redundant sorting signals that function, once Pex3 is inserted into the ER, to prevent it from following the secretory pathway, and that are required for its transport to peroxisomes. These intra-ER sorting signals are sufficient to direct an ER resident membrane protein to the pER subdomain. We show that the N-terminal segments of yeast, human and *Drosophila* Pex3 are functionally interchangeable. Additionally, we found that the transmembrane segment of Pex3 contributes to efficient transport to peroxisomes. We show that all peroxisomes in wild type cells receive newly synthesized Pex3 and a variety of other PMPs. Our findings support a model whereby Pex3 is inserted into the ER membrane where it is sorted to an ER subdomain dependent on its N-terminal signals. From there its efficient transport to peroxisomes requires the transmembrane segment.

## Results

### The basic cluster in the N-terminal segment of ScPex3 is not required for function

Pex3 is a type III integral peroxisomal membrane that reaches peroxisomes via the ER (Höhfeld et al., 1991; Faber et al., 2002; Hoepfner et al., 2005; Tam et al., 2005; Kragt et al., 2005; Motley and Hettema, 2007; Thoms et al., 2012). We argued that for Pex3 to reach peroxisomes efficiently, it will need a signal for its targeting and insertion into the ER membrane and then a second signal for its segregation away from the secretory pathway to the pER. An additional signal may be required for its exit from the pER.

A cluster of basic amino acid residues amino terminal to the transmembrane segment is found in all Pex3 orthologues (Fig. 1B). Recently, this basic cluster was suggested to be part of a signal-anchor-like sequence for targeting and insertion of ScPex3 into the ER (Thoms et al., 2012). We mutated this cluster of amino acids from RHRGK to SASAS in ScPex3-GFP behind its own promoter and expressed it in *pex3*Δ cells also expressing the peroxisomal matrix marker HcRed-PTS1 (Fig. 1D). *pex3*Δ cells lack peroxisomal structures and therefore mislocalise the peroxisomal marker *Hc*Red-PTS1 to the cytosol (Fig. 1D). Since peroxisomes are the only site of fatty acid beta-oxidation in yeasts, *pex3*Δ cells are unable to grow on oleate as sole carbon source. Both versions of Pex3-GFP complement the *pex3*Δ cells' defect for growth on oleate, restore the formation of peroxisomes and localise to peroxisomes (Fig. 1C,D). These observations indicate that the basic cluster is not essential for transport or function of *Sc*Pex3.

### The ER targeting signal of *Sc*Pex3

**Fig. 2.**
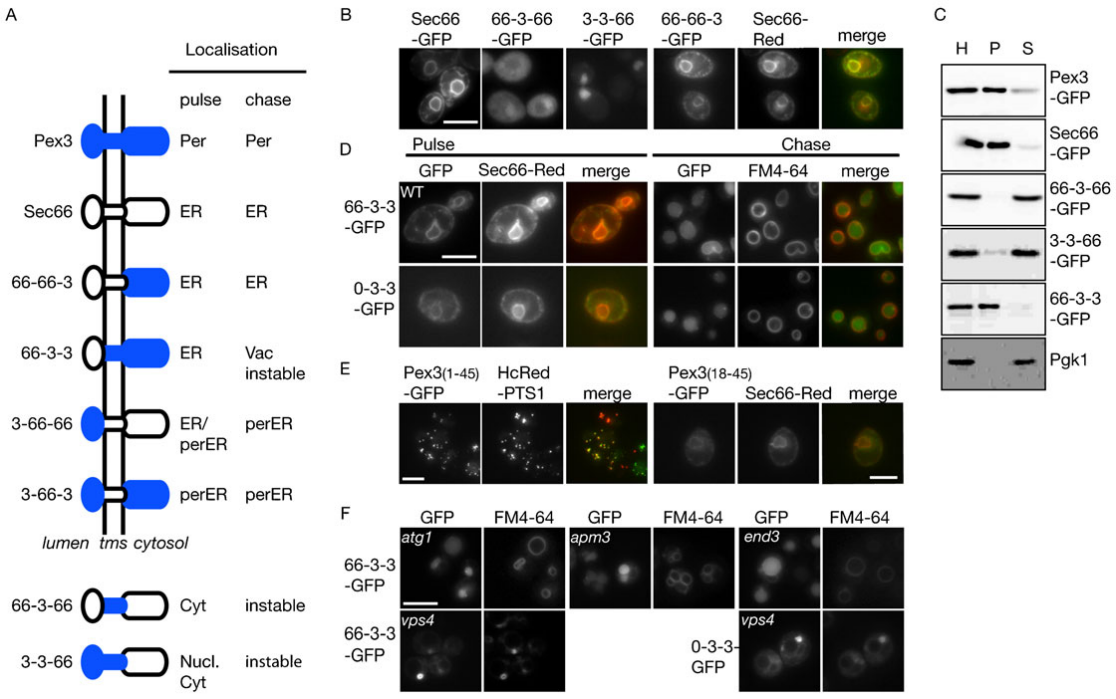
Distribution of Pex3-Sec66-GFP chimeras and Pex3-GFP deletion constructs. (**A**) Overview of the intracellular distribution of Pex3-Sec66 chimeras. Chimeras were expressed by induction of the *GAL1* promoter for 45–60 min (pulse) and switched to glucose medium (chase) 2–3 h before imaging. Per, peroxisome;Vac, vacuole; perER, peroxisomal ER subdomain; cyt, cytosol. (**B**) Fluorescence microscopy of Sec66-GFP and the chimeras after pulse. (**C**) Immunoblotting of a subcellular fractionation of wild type cells expressing indicated chimeras and control proteins using anti-GFP. Anti-Pgk1 was used as cytsolic control in all fractionations but is shown only for the fractionation of 66-3-3-GFP transformant. H, Homogenate; P, 20,000 *g* pellet; S, 20,000 *g* supernatant. (**D**) Fluorescence microscopy pulse-chase analysis of Pex3-GFP versions where the N-terminal segment was exchanged for that of Sec66 (66-3-3-GFP) or deleted (0-3-3-GFP). After initial labelling of the ER (pulse 50 min), the proteins are sorted to vacuoles identified by FM4-64 (chase 2 h). (**E**) The first 45 amino acid residues of Pex3 are sufficient to direct GFP to peroxisomes identified by HcRed-PTS1 (pulse 1 h). Deletion of the N-terminal tail (resulting in Pex3_18–45_-GFP) affects sorting to peroxisomes but not labelling of the ER (pulse 1 h). (**F**) Distribution of 66-3-3-GFP and 0-3-3-GFP in various mutants. Indicated Expression was induced for 1 h and chased for 2 h. Vacuoles were stained with FM4-64. FM4-64 enters cells by endocytosis and reaches the vacuole via the prevacuolar structures. Note the low level of FM4-64 in *end3*Δ cells and the accumulation in prevacuolar structures in *vps4*Δ cells. Colocalisation studies of chimeras with Sec66-Red were performed in diploid cells, hence the increase in cell size. Scale bars: 5 µm.

To investigate the targeting and sorting signals in Pex3, we constructed chimeras of Pex3 with the ER resident type III membrane protein Sec66. Sec66 is dynamically retained in the ER by rapid retrieval from the Golgi in an Rer1/COPI-dependent sorting step. Retrieval is dependent upon recognition of the Sec66 transmembrane segment by Rer1 (Sato et al., 2003). Chimeras were designed so that N-terminal (amino acids 1–17), transmembrane (amino acids 18–39) or cytosolic (amino acids 40–end) segments of Pex3 (Fig. 1B) were exchanged between the two proteins, so that the overall architecture was retained (Fig. 2A). We use a simplified nomenclature to describe the chimeras. For instance, the N-terminal, lumenal part of Sec66 fused to the transmembrane segment of Pex3 followed by the Pex3 cytoplasmic domain is described as 66-3-3 (Fig. 2A). Expression was regulated by the conditional *GAL1* promoter and induced by growth on galactose medium for 40–60 minutes (pulse) followed by a chase of 3 h on glucose-containing medium.

The transmembrane segment of Sec66 is sufficient for ER targeting and insertion (Sato et al., 2003). As expected, the chimera 66-66-3 colocalises with Sec66 to the ER (Fig. 2B). However, the chimeras 66-3-66 and 3-3-66 are both unstable although they can be detected in the cytosol and nucleus, respectively (Fig. 2B). Indeed, subcellular fractionation shows that 66-3-66 and 3-3-66 are not associated with the 20,000 *g* organellar pellet that contains peroxisomes and ER membranes (where Pex3-GFP and Sec66-GFP fractionate), but instead cofractionate with the cytosolic marker Pgk1 (Fig. 2C). The chimera 66-3-3 inserts into the ER (Fig. 2D). Taken together, these observations suggest that the transmembrane segment together with the cytosolic domain contain the information for Pex3 to target and insert into the ER membrane and that the N-terminal segment is not required. Indeed, newly synthesised Pex3-GFP lacking its N-terminal segment (0-3-3-GFP) localises to the ER (Fig. 2D).

The N-terminal 45 amino acid residues of Pex3 are necessary and sufficient for targeting to peroxisomes via the ER (Halbach et al., 2009; Tam et al., 2005) (Fig. 2E). Since the N-terminal segment is not required for targeting to the ER, we generated Pex3_18–45_-GFP and found it localised to the ER after a pulse (Fig. 2E). We conclude that the transmembrane segment followed by six amino acid residues on the cytosolic side of the membrane contains an ER targeting signal.

The absence of the N-terminal segment of Pex3 (66-3-3, 0-3-3) results in proteins that mislocalise to the vacuole (Fig. 2D). FM4-64 enters cells via endocytosis and reaches the vacuolar membrane via endosomes (Vida and Emr, 1995). We tested the route 66-3-3-GFP takes to reach vacuoles by genetically blocking known routes. Blocking autophagy, endocytosis or direct transport from the Golgi to the vacuole in *atg1Δ*, *end3Δ* and *apm3Δ* cells, respectively, did not prevent 66-3-3-GFP reaching vacuoles (Fig. 2F). When transport from endosomes to the vacuole is blocked by deletion of *VPS4*, both FM4-64 and the chimera accumulate in the exaggerated pre-vacuolar or endosomal compartment typical of this mutant. This indicates that 66-3-3 travels from the ER via the Golgi and endosomes to the vacuole. 0-3-3-GFP transport to vacuoles is also blocked only in *vps4Δ* cells (Fig. 2F).

We conclude that insertion of Pex3 into the ER depends on a signal that resides within the transmembrane segment and the amino acid residues immediately following it on the cytoplasmic side of the membrane. Moreover, the N-terminal segment of Pex3 prevents Pex3 from following the secretory pathway to the vacuole, and is required for its sorting from ER to peroxisomes.

### The N-terminal segment of Pex3 sorts an ER membrane protein to the peroxisomal ER subdomain

**Fig. 3.**
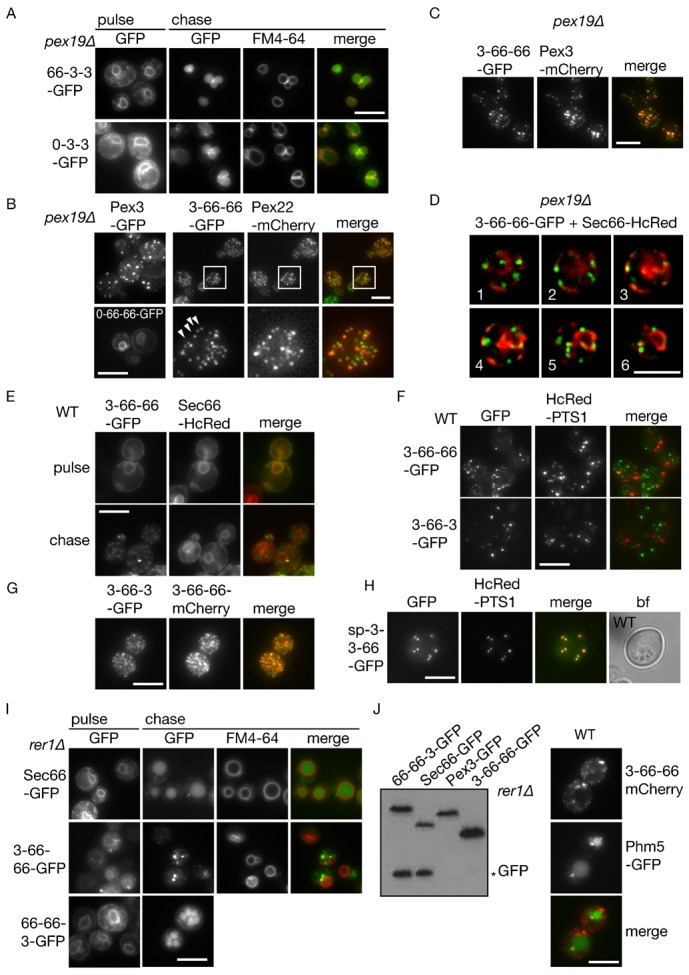
The N-terminal segment of Pex3 is necessary and sufficient to sort an ER protein to the peroxisomal ER subdomain. (**A**) Pulse-chase analysis of the distribution of 66-3-3-GFP and 0-3-3-GFP in *pex19*Δ cells. Both GFP fusions sort via ER to the vacuole identified by FM4-64 in *pex19Δ* cells. Single focal plane images are presented. (**B**,**C**) The N-terminal segment of Pex3 directs an ER membrane protein (3-66-66-GFP) to the punctate peroxisomal ER subdomain in *pex19*Δ cells identified by Pex22-mCherry or Pex3-mCherry. Pex3-GFP and 0-66-66-GFP are included as controls. The fluorescent structures are mobile and fixation with formaldehyde immobilizes only some of puncta. White squares are magnified in lower panel. Arrows indicate colocalising puncta. (**D**) *pex19*Δ cells coexpressing 3-66-66-GFP with Sec66-HcRed reveal close association of 3-66-66-GFP with the ER. Image Z stack is shown after deconvolution. Numbers indicate individual slices in the stack. (**E**) 3-66-66-GFP localizes to the Sec66-HcRed-labelled ER during the pulse, and chases to a punctate compartment in WT cells. (**F**) 3-66-66- and 3-66-3-GFP do not localize to peroxisomes in wild type cells labelled with constitutively expressed HcRed-PTS1. (**G**) 3-66-66-mCherry and 3-66-3-GFP were expressed for 1 h on galactose and chased for 2 h on glucose. (**H**) Addition of invertase signal peptide (SP) restores transport of 3-3-66-GFP to peroxisomes labelled with HcRed-PTS1. SP-3-3-66-GFP was induced for 3 h on galactose medium. (**I**) The N-terminal segment of Pex3 prevents Sec66 from being transported to the vacuole. Pulse-chase analysis of the distribution of indicated GFP fusions in *rer1*Δ cells. Vacuoles are labelled with FM4-64. Single focal plane images are presented. (**J**) Immunoblotting of GFP fusion proteins induced on galactose medium for 2 h and chased for 3 h on glucose medium. Asterisk indicates the relatively stable GFP breakdown product typically produced when GFP fusions enter the vacuole. On the right, fluorescence panels indicate that expression of 3-66-66 does not interfere with targeting of a vacuolar membrane protein. Phm5-GFP was co-expressed with 3-66-66-mCherry in WT cells. Scale bars: 5 µm.

Pex3 is sorted via a subdomain of the ER to peroxisomes. In *pex19*Δ cells, Pex3-GFP accumulates in this ER subdomain, as exit from the ER requires Pex19 (Agrawal et al., 2011; Hoepfner et al., 2005; Lam et al., 2011; Tam et al., 2005). In contrast, 66-3-3-GFP and 0-3-3-GFP are initially distributed throughout the ER, as indicated by the striking perinuclear ER staining, and chase to the vacuole in both wild type (Fig. 2D) and *pex19*Δ cells (Fig. 3A). This suggests that the N-terminal segment of Pex3 is necessary for sorting Pex3 to the pER subdomain. The pER resembles Pex3-GFP puncta in close association with the ER (Hoepfner et al., 2005).

We generated 3-66-66-GFP and expressed it in *pex19*Δ cells with either Pex3-mCherry or Pex22-mCherry, which has been shown to colocalise with Pex3 in the peroxisomal ER in *pex19*Δ cells (Halbach et al., 2009). Whereas 0-66-66-GFP localizes throughout the ER (Fig. 3B), the chimera 3-66-66-GFP colocalised with Pex22-mCherry and Pex3-mCherry in *pex19*Δ cells (Fig. 3B,C). 3-66-66-GFP puncta are closely associated with the ER (Fig. 3D). This indicates that the N-terminal segment of Pex3 is sufficient to target an ER resident protein to the pER subdomain.

At early time points during the pulse, 3-66-66 colocalised with the ER marker Sec66-HcRed in wild type cells, and during the chase, the labelling changed to a punctate pattern (Fig. 3E). Although 3-66-66 reaches the pER in *pex19*Δ cells (Fig. 3B), it does not reach peroxisomes in wild type cells (Fig. 3F). Interestingly, the chimera 3-66-3 also does not reach peroxisomes in wild type cells (Fig. 3F), but colocalises with 3-66-66 (Fig. 3G). In order to test whether the transmembrane segment is required for the subsequent transport from the pER to peroxisomes, we constructed the chimera 3-3-66 fused to a signal peptide (SP-3-3-66-GFP). We added the signal peptide as 3-3-66 lacks a functional ER targeting signal (Fig. 2B). This chimera now reaches peroxisomes (Fig. 3H).

We conclude that the Pex3 N-terminal segment is sufficient for sorting an ER membrane protein to the pER. Moreover, the transmembrane segment of Pex3 contributes to transport from the pER to peroxisomes.

Subsequently, we tested whether the N-terminal segment of Pex3 can prevent a membrane protein from following the secretory pathway. The ER localization of Sec66 requires Rer1-dependent retrieval from the Golgi to prevent it from reaching vacuoles via endosomes (Sato et al., 2003). Indeed, newly synthesized Sec66 first localizes to the ER, but subsequently (during the chase) accumulates in the vacuole in *rer1Δ* cells (Fig. 3I). Delivery to the vacuole of GFP fusion proteins results in their breakdown, but the relative protease resistance of GFP allows its delivery to the vacuole to be monitored by Western blot detection of the GFP breakdown product. As expected, in *rer1Δ* cells, the breakdown product typical of GFP fusion accumulates besides the full length Sec66-GFP (Fig. 3J). The same result was obtained when the cytosolic domain of Sec66 was exchanged for that of Pex3, resulting in the chimera 66-66-3-GFP (Fig. 3I,J). However, the chimera 3-66-66-GFP behaved differently. A pulse of 3-66-66-GFP expression in *rer1Δ* cells resulted in weak ER labelling plus puncta (Fig. 3I). No vacuolar labelling was observed during the chase and the GFP breakdown product was absent (Fig. 3J). Expression of 3-66-66 does not interfere with trafficking of the vacuolar polyphosphatase Phm5 that is synthesized as a transmembrane precursor protein (Fig. 3J). This means that the N-terminus of Pex3 prevents 3-66-66 from following the secretory pathway in *rer1Δ* cells. Therefore, the N-terminal segment contains information that diverts a membrane protein in the ER away from the secretory pathway into the peroxisomal sorting pathway.

### Two signals in the N-terminal segment sort Pex3 to the pER

**Fig. 4.**
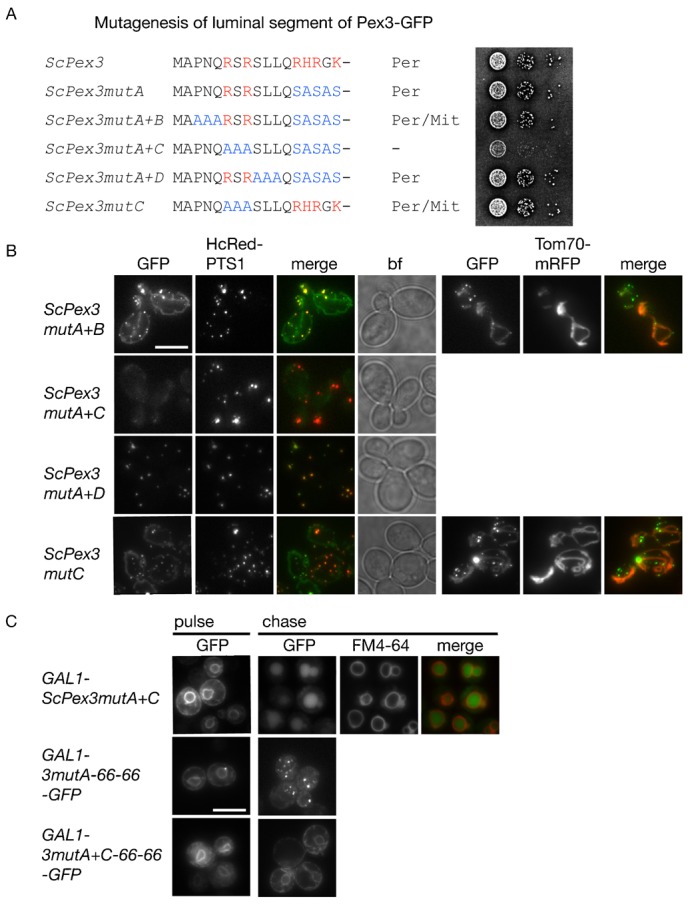
Mutants that affect sorting of Pex3 to the peroxisomal ER. (**A**) Overview of mutations in the N-terminal segment of Pex3 and the effect on subcellular localization and function. Basic residues indicated in red, mutations in blue. Per, peroxisome; Mit, mitochondrion; –, undetectable. Right hand panel shows serial dilution of pex3 cells transformed with Pex3-GFP and mutant versions under control of the *PEX3* promoter grown for 7 days on oleate medium. (**B**) Distribution of Pex3-GFP mutants in WT cells constitutively expressing HcRed-PTS1. Bf, bright field. ScPex3mutA+B-GFP and ScPex3mutC-GFP were co-expressed with the mitochondrial outer membrane marker Tom70-mRFP. (**C**) Pulse-chase analysis of the distribution of Pex3mutA+C-GFP and the chimeras 3mutA-66-66-GFP and 3mutA+C-66-66-GFP in WT cells. During pulse (1 h galactose medium) GFP fusions label the ER. During chase (2 h glucose), Pex3mutA+C-GFP sorts to the vacuole, as identified by FM4-64 whereas the chimera 3mutA+C-66-66-GFP fails to sort to the peroxisomal ER subdomain and stains the ER. The chimera 3mutA-66-66-GFP displays a punctate labelling pattern. Scale bars: 5 µm.

Since the evolutionarily conserved basic cluster of amino acid residues in the N-terminal segment of *Sc*Pex3 is not essential for its transport but has been reported to be important for transport of Pex3 orthologues, we tested whether an additional sorting signal is present in this segment of *Sc*Pex3. We mutagenised the N-terminal segment already mutated in the positive cluster in Pex3-GFP (ScPex3mutA) expressed under control of its own promoter in wild type cells (Fig. 4A). When we mutated 3PNQ5 to 3AAA5 (ScPex3mutA+B), the protein still reached peroxisomes but some mitochondrial labelling was also observed (Fig. 4B). Mutation of 6RSR8 to AAA (ScPex3mutA+C) resulted in a version of Pex3 that was undetectable whereas 9SLL11 to AAA (ScPex3mutA+D) did not affect Pex3 sorting (Fig. 4B). All these mutants restored growth on oleate when expressed in *pex3*Δ cells except for ScPex3mutA+C (Fig. 4A).

Mutation of 6RSR8 in an otherwise wild type *PEX3* gene (ScPex3mutC) did not block sorting to peroxisomes although some mitochondrial labelling was observed (Fig. 4B).

To investigate the instability of ScPex3mutA+C, we expressed it from the *GAL1* promoter and performed a pulse-chase experiment. The mutant targeted to the ER during the pulse, and was subsequently sorted to the vacuole during the chase (Fig. 4C). These results indicate that the N-terminal segment of Pex3 contains two signals that can act independently in sorting Pex3 to peroxisomes. Introduction of mutation A in 3-66-66-GFP did not affect its intra-ER sorting in wild type cells. However, the chimera containing mutations A+C was no longer sorted during pulse or chase but instead was present throughout the ER. In *rer1*Δ cells, it ended up in the vacuole (not shown). We conclude that the N-terminal segment of Pex3 contains two independently acting signals that mediate sorting to the pER.

### Pex3 sorting is evolutionarily conserved

**Fig. 5.**
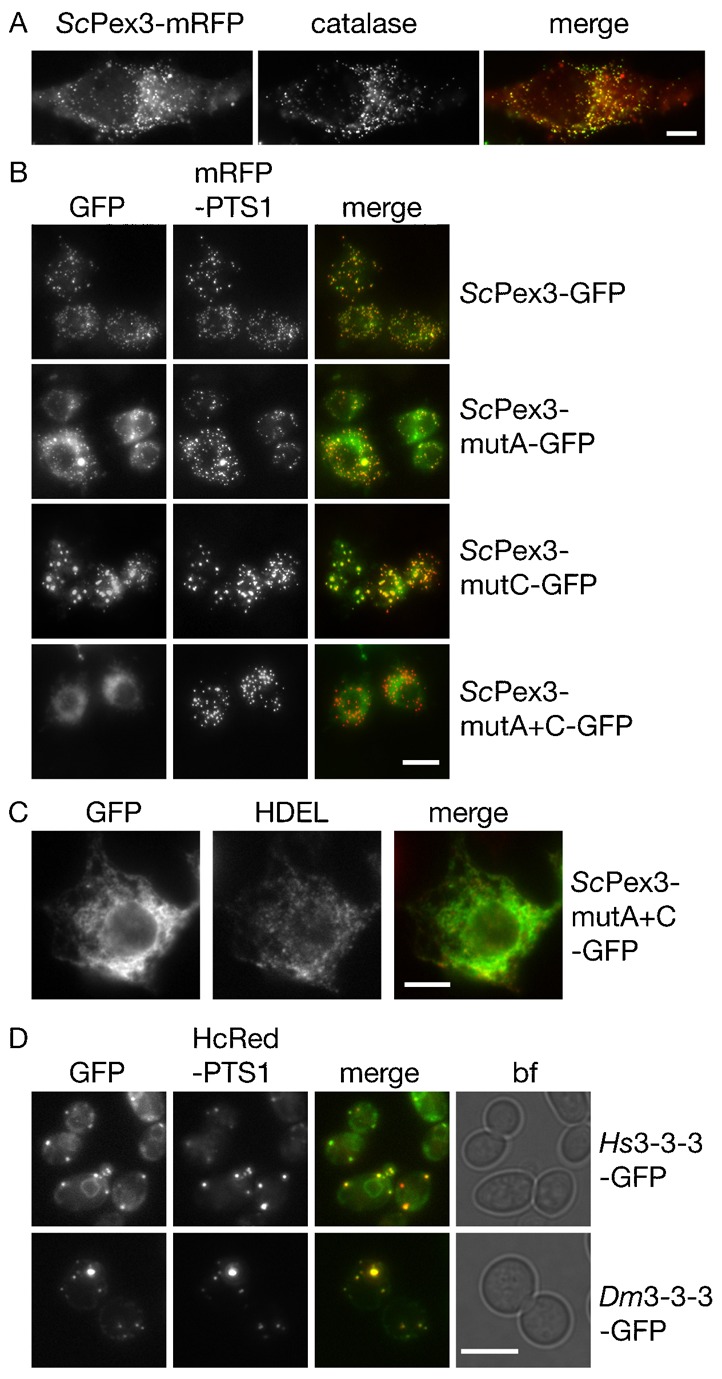
Evolutionary conservation of Pex3 transport signals. (**A**) ScPex3-GFP localizes to human peroxisomes. Hela cells transiently transfected with ScPex3-GFP under control of the CMV promoter were processed for immunofluorescence with anti-catalase. Scale bar: 10 µm. (**B**) Distribution of *Sc*Pex3-GFP and mutants thereof in *Drosophila* cells. SR2+ cells expressing mRFP-PTS1 were transiently transfected with the various *Sc*Pex3-GFP versions under control of the conditional metallothionein promoter. Expression was induced by addition of 100 µM CuSO_4_ 15 h before imaging. Scale bar: 10 µm. (**C**) Immunofluorescence of S2R+ cells expressing *Sc*Pex3mutA+C-GFP using monoclonal anti-HDEL detected by goat anti-mouse Rhodamine. Scale bar: 5 µm. (**D**) Human and *Drosophila* N-terminal segment of Pex3 function in *S. cerevisiae*. The N-terminal segment of ScPex3 was replaced by the Human (*Hs*) or *Drosophila melanogaster* (*Dm*) Pex3 segment and expression was induced by growth on galactose medium for 1 h in *S. cerevisiae* cells expressing *Hc*Red-PTS1. Scale bar: 5 µm.

When expressed in mammalian and *Drosophila* S2R+ cells, *Sc*Pex3 colocalised with the peroxisomal marker protein (Fig. 5A,B, top panel). Furthermore, localisation of ScPex3-GFP to peroxisomes in *Drosophila* S2R+ cells requires the presence of either RSR or RHRGK in the N-terminal segment, as only the double mutant failed to be sorted to peroxisomes in these cells (Fig. 5B). This mutant appears to be trapped in the ER of S2R+ cells, as it substantially overlaps with the HDEL-containing tubular compartment (Fig. 5C).

We tested whether the N-terminal segment of human and *Drosophila* Pex3 can replace the *S. cerevisiae* N-terminal segment. This is indeed the case: chimers containing the human or *Drosophila* N-terminal segment are able to target Pex3 to peroxisomes in wild type yeast (Fig. 5D). We conclude that the mechanism for sorting Pex3 to the peroxisomal ER is evolutionarily conserved.

### PMPs are transported to existing peroxisomes

Previously, we have shown that existing peroxisomes receive newly synthesized Pex3 (Motley and Hettema, 2007). However, two recent papers report PMPs traffic via the ER to form new peroxisomes (van der Zand et al., 2010; van der Zand et al., 2012). According to van der Zand et al., PMPs bud off the pER forming distinct vesicles which do not fuse with existing (PTS1-containing) peroxisomes, but fuse heterotypically with each other to generate a new functional peroxisome which then becomes import-competent for PTS1-containing matrix proteins (van der Zand et al., 2012). We induced expression of Pex3 for up to 45 minutes in wild type cells and noticed that at the earliest detection points, all existing (i.e. PTS1-containing) peroxisomes received material. This replicates our previous observations (Motley and Hettema, 2007) and means that all newly synthesized Pex3 associates with peroxisomes.

**Fig. 6.**
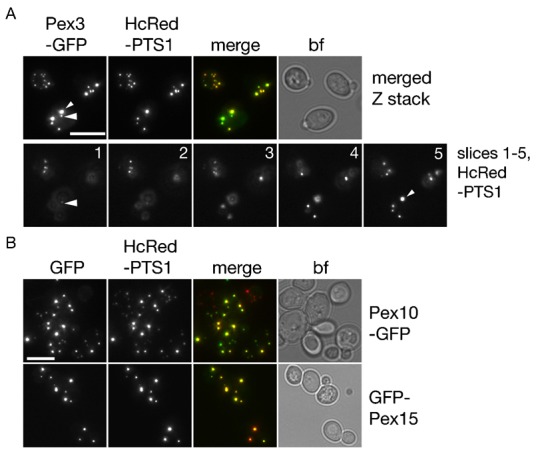
Peroxisomes receive newly synthesized PMPs in cells blocked in peroxisome breakdown. All peroxisomes identified by *Hc*Red-PTS1 received newly synthesised Pex3-GFP (**A**), Pex10-GFP and GFP-Pex15 (**B**). Expression was induced for 40 min on galactose medium in *atg36*Δ cells. Large arrow head indicates Pex3-GFP punctum appearing to lack any HcRed-PTS1 colocalisation in merged Z stack. Small arrow head is used as reference point. In separate slices of the Z-stack, colocalisation is observed. Numbers indicate slice number. Bf, bright field. Scale bars: 5 µm.

Although under our experimental conditions no pexophagy is expected (Motley et al., 2012a; Motley et al., 2012b), we wanted to rule out an alternative explanation for all peroxisomes receiving newly synthesized Pex3, that peroxisomes are turned over and rapidly formed *de novo*. To test this, we induced expression of the PMPs Pex3-GFP, Pex10-GFP and GFP-Pex15 in cells blocked in peroxisome turnover (*atg36*Δ cells) also expressing the peroxisomal luminal marker HcRed-PTS1. We observed that all HcRed-PTS1 containing peroxisomes labelled with the newly synthesized PMPs (Fig. 6). Since peroxisomes do not fuse (Motley and Hettema, 2007), we conclude that newly synthesized PMPs are transported to existing peroxisomes.

## Discussion

Pex3 is required at an early stage of peroxisome formation. Loss of function mutations in Pex3 result in a complete absence of peroxisomal structures and in humans this leads to the lethal Zellweger syndrome.

ScPex3 has been shown to be a single-spanning membrane protein of type III topology (N_exo_/C_cyt_) (Höhfeld et al., 1991; Kragt et al., 2005). After being inserted into the ER, it is sorted to a subdomain of the ER and from here is trafficked to peroxisomes (Hoepfner et al., 2005; Tam et al., 2005). The N-terminal 45 amino acid residues of ScPex3 contain the short luminal (17 amino acids) segment and the transmembrane segment followed by a few amino acid residues. This Pex3 domain has been shown to be necessary and sufficient to direct Pex3 to the peroxisomal membrane (Tam et al., 2005) and can be exchanged with the N-terminal domain of another type III PMP, Pex22 (Halbach et al., 2009). However, the signals in Pex3 that mediate insertion into the ER membrane, intra-ER sorting and transport to peroxisomes have not been identified. Previous deletion analyses have not led to identification of the signals required for the individual steps in the targeting pathway because there is overlap and interdependence between signals. We used a combination of approaches to identify the targeting and sorting signals in Pex3, including deletion analyses, site directed mutagenesis and chimera construction with the type III ER protein Sec66. The use of a conditional expression system allowed us to study the fate of newly synthesized Pex3 chimeras during pulse-chase experiments even if these mutants were instable.

### Identification of the ER targeting signal

Peroxisome formation requires the activity of Sec61, and Pex3 was suggested to contain a signal anchor-like sequence consisting of the evolutionarily conserved positive cluster followed by the transmembrane segment (Thoms et al., 2012). In line with this suggestion was the observation that introduction of two basic residues into the transmembrane segment resulted in mislocalisation to the cytosol. However, we did not observe a requirement for the positive cluster preceding the transmembrane segment for transport to peroxisomes or for Pex3 function. Furthermore, deletion of the complete N-terminal segment did not affect Pex3 transport to the ER (Fig. 2D). In contrast, the chimeras 3-3-66 and 66-3-66 do not associate with ER or peroxisomes (Fig. 2B,C). Furthermore, a construct containing the transmembrane segment followed by 6 amino acids of the cytosolic domain of Pex3 targeted to the ER (Fig. 3E, right hand panel). The features of this signal resemble those of a reverse signal anchor sequence (Goder and Spiess, 2001).

### Intra-ER sorting signals

The ER is a complex organelle consisting of a multitude of subdomains (Levine and Rabouille, 2005) each with their own functions and specific protein composition. The limited number of signals that have been described to mediate intra-ER sorting reside in transmembrane segments or cytosolic sequences (Lynes and Simmen, 2011; Ronchi et al., 2008; and references therein).

Pex3 passes through a subdomain of the ER in transit to peroxisomes. This subdomain is hard to detect in wild type cells, but is readily visualized in mutants such as *pex19* that are blocked in exit of Pex3 from the ER (Hoepfner et al., 2005; Tam et al., 2005). Our data show that the N-terminal segment of Pex3 is both necessary and sufficient to sort an ER membrane protein to the peroxisomal ER subdomain in *pex19* and wt cells (Fig. 3B,D). We identified two intra-ER sorting signals in the Pex3 N-terminal segment, including the evolutionarily conserved basic cluster just preceding the transmembrane segment. Mutation of both signals in ScPex3 resulted in its breakdown as a consequence of missorting to the vacuole. We found that the ScPex3 sorting signals are active in *Drosophila* and human cells and that the N-terminal segments of *Drosophila* and human Pex3 are active in peroxisomal targeting when replacing the N-terminal segment of *Sc*Pex3 (Fig. 5). We conclude that the mechanism of intra-ER sorting of Pex3 is evolutionarily conserved and is dependent on signals in the N-terminal segment of Pex3. Both signals in ScPex3 contain positively charged residues and it may be that the net positive charge of the luminal segment is sufficient for sorting.

Topology studies indicate that the N-terminus of newly synthesized Pex3 is exposed to the lumen of the ER (Kragt et al., 2005; Thoms et al., 2012) and therefore we propose that intra-ER sorting of Pex3 is dependent upon luminal recognition of its sorting signals.

Pex22 is another type III PMP that traffics via the ER and the peroxisomal ER to peroxisomes (Halbach et al., 2009). The ScPex22 N-terminal domain can functionally replace that of ScPex3 (Halbach et al., 2009) and contains several positive amino acid residues including the motif RSR also found in ScPex3. It is tempting to speculate that the sorting mechanism of Pex3 may be shared by some other PMPs, but this needs further testing.

### Transport to peroxisomes

Multiplication of peroxisomes by growth and division has been the accepted model since 1985 (Lazarow and Fujiki, 1985). However, later studies revealed that peroxisomes can form de novo from the ER (Hoepfner et al., 2005; Tam et al., 2005; Kim et al., 2006; Toro et al., 2009; van der Zand et al., 2010; van der Zand et al., 2012) although this seems not to be the dominant mechanism of peroxisome multiplication (Delille et al., 2010; Huybrechts et al., 2009; Kim et al., 2006; Motley and Hettema, 2007; Nagotu et al., 2008b). A model proposing that the ER supplies membrane constituents to existing peroxisomes was based on the observation that at least some newly synthesized PMPs reach existing peroxisomes via the ER (Mullen and Trelease, 2006; Motley and Hettema, 2007). However, two recent studies propose that peroxisomes in wild type *S. cerevisiae* cells multiply by de novo formation (van der Zand et al., 2010; van der Zand et al., 2012).

A crucial difference between the two models is that most if not all (PTS1-containing) peroxisomes should receive newly synthesized PMPs according to the growth and division model whereas only preperoxisomal (non-PTS1-containing) vesicles receive PMPs according to the de novo formation model. When reinvestigated, we were able to confirm that all (PTS1-containing) peroxisomes receive newly synthesized Pex3, even in cells with a block in peroxisome turnover. Therefore, pre-existing peroxisomes receive Pex3 under our assay conditions. This conclusion is in line with our previous observations and that of others that yeast peroxisomes multiply by fission (Menendez-Benito et al., 2013; Nagotu et al., 2008a; Nagotu et al., 2008b) and form de novo only in cells temporarily devoid of peroxisomes (Motley and Hettema, 2007).

Our analysis has uncovered the signals within Pex3 required for the various steps of its transport to peroxisomes. We have generated versions of Pex3 that are blocked at each stage along its transport pathway. The availability of these versions of Pex3 will be instrumental in unravelling further the mechanism of intra-ER sorting and Pex3 transport to peroxisomes as well as the machinery required en route. These tools may be of wider use: Pex3 has also been shown to localize first in the ER and subsequently in newly formed peroxisomes of human fibroblasts (Toro et al., 2009), and we show that the intra-ER sorting signals in Pex3 are evolutionarily conserved.

## Materials and Methods

### Yeast strains and media

The yeast strains used in this study BY4742 *Matα; his3Δ1, leu2Δ1,lysΔ0, ura3Δ10* and its derivatives were obtained from EUROSCRAF; BY4742 *pex3*::KanMX, BY4742 *pex19*::KanMX, BY4742 *apm3*::KanMX, BY4742 *vps4*::KanMX, BY4742 *end3*::KanMX and the BY4741 *Mat A; his3Δ1, leu2Δ0, met15Δ0, ura3Δ0* derivatives BY4741 *rer1*::KanMX, BY4741 *atg1*::KanMX. Yeast cells were grown at 30°C in either of the following mediums: YPD media (1% yeast extract, 2% peptone, 2% glucose), minimal media (YM2) for the selection of the uracil prototrophic marker (carbon source, 0.17% yeast nitrogen base without amino acids, 0.5% ammonium sulphate, 1% casamino acids), or minimal media (YM1) for the selection of other prototrophic markers (carbon source, 0.17% yeast nitrogen base without amino acids, 0.5% ammonium sulphate). Regarding the carbon sources, glucose, raffinose and galactose were added to 2% (w/v). The appropriate amino acids were added to minimal media as required. Oleate plates contained 0.67% yeast nitrogen base without amino acids, 0.5% ammonium sulphate, 0.1% yeast extract, 0.1% oleate (v/v), 0.25% Tween-40® (v/v), 2% agar, and amino acids as needed. For pulse-chase experiments, a preculture grown on minimal glucose medium was transferred to minimal raffinose medium and incubated overnight. Next morning, cells were 1:10 diluted in prewarmed 2% galactose medium at (pulse) and incubated at 30°C for the time indicated in the text and subsequently spun down at 3.500 rpm for 2 minutes in an eppendorf centrifuge and the cell pellet was transferred to prewarmed glucose medium (start of chase).

### Plasmids

Yeast expression plasmids were based on the parental plasmids ycplac33 and ycplac111 (Gietz and Sugino, 1988). The majority of constructs used in this study were generated by gap repair in yeast (Uetz et al., 2000). The ORF or parts thereof of interest was amplified by PCR. The 5′ ends of the primers included 18 nt extensions identical to plasmid sequences flanking the intended insertion site, to enable repair of gapped plasmids by homologous recombination. This way of construction allows for fusion of protein encoding fragments without the introduction of restriction sites between the fragments. Mutations in the N-terminal segment of Pex3 were introduced by PCR using primers containing the mutations and the gap repair methodology. For expression of genes under control of their endogenous promoter, 500 nt upstream from the ORF were included. Galactose-inducible constructs contained the *GAL1/10* intergenic region. Constitutive overexpression was achieved by use of the *Tpi1* promoter region. All constitutive expression constructs contain the *PGK1* terminator. *GAL1/10*-containing plasmids contain the *MFA2* terminator (Motley and Hettema, 2007). DNA sequence was confirmed of all construct generated in this study. The constitutive expression constructs for HcRED-PTS1 and GFP-PTS1 have been described previously (Motley and Hettema, 2007; Munck et al., 2009). We used GFPS65T and mRFP for tagging.

### Fluorescence microscopy

Live cells were analysed with an Axiovert 200M microscope (Carl Zeiss, Inc.) equipped with Exfo X-cite 120 excitation light source, band-pass filters (Carl Zeiss, Inc. and Chroma), and alpha Plan-Fluar 100×/1.45 NA or Plan Apochromat 63×/1.4 NA objective lens (Carl Zeiss, Inc.) and a digital camera (Orca ER; Hamamatsu). Image acquisition was performed using Openlab and Volocity software (PerkinElmer). Fluorescence images were routinely collected as 0.5 µm Z-Stacks and merged into one plane in Volocity and processed further in Photoshop (Adobe) where only the level adjustment was used. On occasions (as indicated in text) images were collected and displayed as single-plane images. Bright-field images were collected in one plane. Deconvolved images were generated using Volocity iterative deconvolution. For colocalisation experiments, cells were fixed with 3.6% formaldehyde for up to 5 minutes. Cells were harvested and resuspended in PBS containing 0.1 M ammonium chloride. Immunofluorescence was performed as described previously (Motley et al., 1994). Anti-HDEL and anti-human catalase were obtained from Abcam.

The vacuolar membrane was stained as previously described (Vida and Emr, 1995).

### Subcellular fractionation and immunoblotting

Subcellular fractionation was performed as described previously (Hettema and Tabak, 2000). For preparation of extracts by alkaline lysis, cells were centrifuged and pellets resuspended in 0.2 M NaOH and 0.2% β-mercaptoethanol and left on ice for 10 min. Soluble protein was precipitated by addition of 5% TCA for a further 10 min. Following centrifugation (13,000 *g*, 5 min, 4°C), soluble protein was resuspended in 10 µl 1 M Tris-HCl (pH 9.4) and boiled in 90 µl 1× SDS-PAGE sample loading buffer for 10 min. Samples (0.25–1 OD_600_ equivalent) were resolved by SDS-PAGE followed by immunoblotting. Blots were blocked in 2% (w/v) fat-free Marvel™ milk in PBS. Tagged proteins were detected using monoclonal anti-GFP (mouse; 1:3,000; Roche). Secondary antibody was HRP-linked anti-mouse polyclonal (goat; 1:4,000; Bio-Rad). Detection was achieved using enhanced chemiluminescence (Biological Industries) and chemiluminescence imaging.

### Growth and transfection of S2R+ and Hela cells

S2R+ cells were grown in Schneider's *Drosophila* Medium containing 10% Newborn Calf Serum at 25°C, 100% humidity. S2R+ cells were transfected with the desired plasmid according to Effectene (Qiagen) protocols. Transfection medium was removed and cells were resuspended in fresh medium. 100 µl of medium containing 1–3×10^5^ cells was placed on a sterile coverslip in a well of a 6-well plate and the cells were allowed to attach for 2–3 hrs. Expression was induced O/N with 100 µM CuSO_4_.

Growth conditions and transfections of Hela cells were performed as described previously (Motley et al., 2003).
